# Gene Cloning, Expression and Activity Analysis of Manganese Superoxide Dismutase from Two Strains of *Gracilaria lemaneiformis *(Gracilariaceae, Rhodophyta) under Heat Stress

**DOI:** 10.3390/molecules17044522

**Published:** 2012-04-16

**Authors:** Ning Lu, Xiaonan Zang, Xuecheng Zhang, Hao Chen, Xiaoting Feng, Lu Zhang

**Affiliations:** Key Laboratory of Marine Genetics and Breeding, Ministry of Education, Ocean University of China, Qingdao 266003, China

**Keywords:** *Gracilaria lemaneiformis*, heat resistance, Mn-SOD, gene expression, SOD activity

## Abstract

Manganese superoxide dismutase (Mn-SOD) plays a crucial role in antioxidant responses to environmental stress. To determine whether Mn-SOD affects heat resistance of *Gracilaria lemaneiformis*, we cloned Mn-SOD cDNA sequences of two strains of this red alga, wild type and cultivar 981. Both cDNA sequences contained an ORF of 675 bp encoding 224 amino acid residues. The cDNA sequences and the deduced amino acid sequences of the two strains shared relatively high identity (more than 99%). No intron existed in genomic DNA of Mn-SOD in *G. lemaneiformis*. Southern blotting indicated that there were multiple copies, possibly four, of Mn-SOD in both strains. Both in the wild type and cultivar 981, SOD mRNA transcription and SOD activity increased under high temperature stress, while cultivar 981 was more heat resistant based on its SOD activity. This research suggests that there may be a direct relationship between SOD activity and the heat resistance of *G. lemaneiformis*.

## Abbreviations

Mn-SOD: manganese superoxide dismutase
W: wild populations of *G. lemaneiformis*
981: cultivar 981 of *G. lemaneiformis*
W-SOD: Mn-SOD of W
981-SOD: Mn-SOD of 981

## 1. Introduction

The edible red seaweed *Gracilaria lemaneiformis* (Gracilariaceae, Rhodophyta) is one of the most important commercial agarophytes and is an important feedstuff for abalone aquaculture [1]. It is also an excellent material to restore a damaged environment that has eutrophic waters [2,3,4]. The wild population of *G. lemaneiformis *grows naturally along the coast of Shandong Province and the preferred temperature for growth is between 11 and 23 °C, thus the growth season is relatively short and the biomass production is low [5], consequently cultivation of heat-tolerant strains of *G. lemaneiformis* has become the goal of many researchers. Cultivar 981, which can endure higher temperatures, was developed as a result of this effort. Phenotypic differences between W and 981 are that the diameter of the algal branches of 981 is smaller than that of W, while the branches and growing points of 981 are much more abundant than those in W. Cultivation experiments have proven the superior features of 981, such as high productivity, good temperature adaptability (from 11 to 26 °C) and high concentration of agar [6,7]. On that basis, *G. lemaneiformis* has been transplanted to the southern coasts of China, thereby prolonging the growth season and increasing production. Despite its superior heat resistance, cultivar 981 cannot naturally survive through the hotter summer along the south coasts, and it is still necessary to transport seed stock from the north shore for large-scale cultures. Therefore, it is very desirable to develop new strains that can endure even higher temperature. Studying the response mechanism of *G. lemaneiformis* to heat stress has become the main concern of many researchers.

The antioxidant enzymatic system is considered to play an important role in resisting heat stress [8]. SOD is a ubiquitous metalloenzyme, capable of converting superoxide radicals to oxygen and hydrogen peroxide. It is the first and most important line of antioxidant enzyme defense systems [9,10]. The level of SOD can reflect plants’ ability to reduce stress [11]. In algae, recent studies have demonstrated that the activities of SOD increased under various environmental stressors [12,13,14,15], and SOD is thus considered to play a critical role in antioxidant protection systems in algae as well. Fe-SOD genes of *Spirulina platensis*, *Nostoc commune* and *Synechocystis* sp. have been cloned and Mn-SOD genes of *Porphyra yezoensis*, *Prulina platensis*, *Chlamydomonas reinhardtii*, *Volvox carteri *f. *nagariensis* and *Haematococcus pluvialis* have also been cloned [16,17]. More and more studies of SOD are being performed in algae; however, there are no such reports for *G. lemaneiformis*.

In order to establish a mechanism for the response of * G. lemaneiformis* to heat stress, the suppression subtractive hybridization (SSH) between the cDNA libraries of *G. lemaneiformis* that lived under common conditions and under heat stress were used to make up the cDNA discrepancy library in our lab. The SSH library was constructed by using the Super SMART™ cDNA synthesis kit (Clontech, Palo Alto, CA, USA) and PCR-Select™ cDNA Subtraction Kit (Clontech). By sequencing and BLAST, 56 differential genes responding to the heat stress were identified initially from the libraries, the EST encoding Mn-SOD was screened from them. Based on the EST sequence, the cDNA and DNA sequences of Mn-SOD for both the wild and cultivar 981 strains of *G. lemaneiformis* were cloned. In this paper, the gene transcription and activity of Mn-SOD in response to heat stress were examined for differences between the two strains.

## 2. Results and Discussion

### 2.1. Cloning and Analysis of Mn-SOD cDNA of G. lemaneiformis

**Figure 1:**
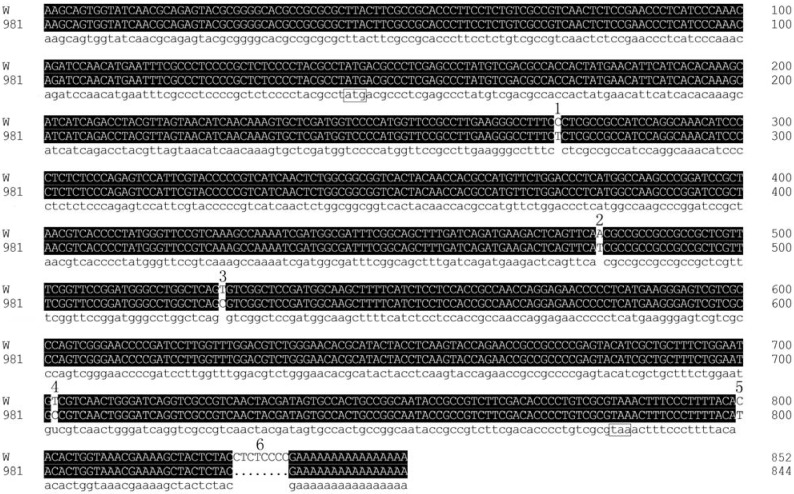
Comparison of cDNA sequences between W-SOD and 981-SOD. Start codon ATG and stop codon TAA are marked with boxes; the six differences are marked with Arabic numbers.

The full-length cDNA of Mn-SOD obtained from the wild strain was 852 bp, with a GC content of 56.4%. The full-length cDNA from cultivar 981 was 844 bp, with a GC content of 56.2%. Alignment of the sequences of the two strains is shown in Figure 1. There were six differences between the two sequences.

Analysis by DNAMAN identified an ORF of 675 bp encoding 224 amino acid residues in both the W-SOD and 981-SOD. For the W-SOD, the 5' UTR contained 109 bp and the 3' UTR 68 bp, whereas for the 981-SOD, the 5' UTR had 109 bp and the 3' UTR 60 bp. The calculated molecular mass of both W-SOD and 981-SOD was 24.2 kD and the isoelectric point of both was 6.97. The identity between the two deduced amino acid sequences was as high as 99.11%. In both strains, the conserved metal-binding domain DVWEHAYY was identified, which is considered a manganese and iron superoxide dismutase signature. This short conserved region includes two of the four ligands of the manganese atom: An aspartate and a histidine [18]. There were only two polymorphisms between W-SOD and 981-SOD—An amino acid replacement at position 124 (Asn/Ile) and another at position 198 (Val/Ala).

**Figure 2:**
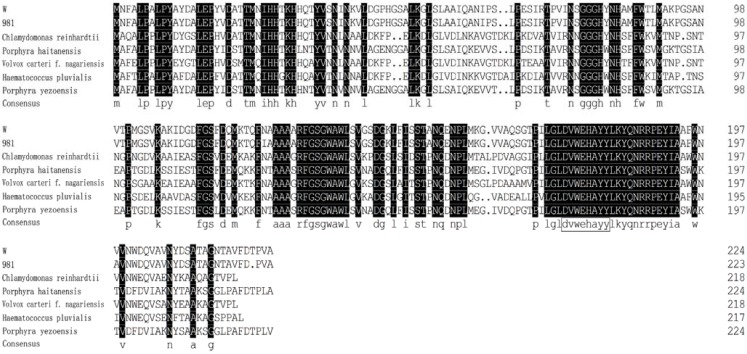
Alignment of Mn-SOD amino acid sequences of *G. lemaneiformis* and other algae. DVWEHAYY is the conserved metal-binding domain. The corresponding proteins are listed as follows: *Porphyra haitanensis* (ACD75820.1), *Porphyra yezoensis *(AAZ75664.1), *Chlamydomonas reinhardtii* (XP_001700058.1), *Haematococcus pluvialis* (AAW69292.1), *Volvox carteri *f. *nagariensis* (XP_002958467.1).

**Figure 3:**
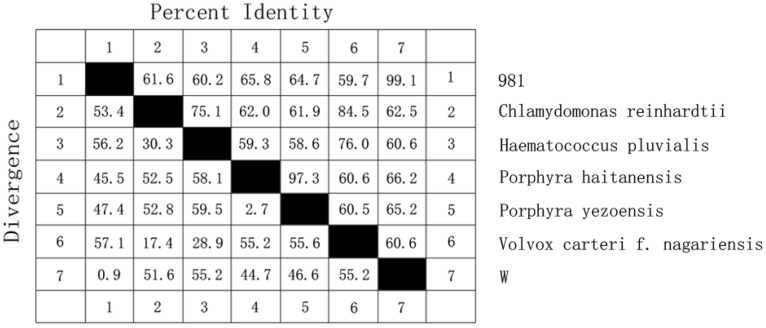
Percent identity and divergence between *G. lemaneiformis* and other algae.

Mn-SOD is a two-domain protein with components of the active site at the N and C terminus of the protein. The deduced amino acid sequences were aligned with other known sequences using the BLAST program [19,20]. The results showed that the iron/manganese superoxide dismutase active site components both in the *N*-terminal and *C*-terminal domains were found as expected. *N*-terminal domain (19–92 aa) is a long alpha antiparallel hairpin and *C*-terminal domain (108–208 aa) is a mixed alpha/beta fold. The BLAST result also indicated that the sequences were similar to Mn-SOD of other algae. Alignment of Mn-SOD amino acid sequences of *G. lemaneiformis* and other algae (red algae: *Porphyra haitanensis* and *Porphyra yezoensis*; green algae: *Chlamydomonas reinhardtii*, *Haematococcus pluvialis* and *Volvox carteri *f. *nagariensis*) are shown in Figure 2. The similarities between them are listed in Figure 3. As displayed in the Figures, Mn-SOD enzymes of *G. lemaneiformis* shared the highest similarity with another red alga, *Porphyra*.

### 2.2. Cloning of the Mn-SOD DNA Sequence

DNA sequences were obtained from both the wild strain and cultivar 981. The sequences were of the same length (675 bp), which was identical to the cDNA ORF. The Mn-SOD gene of *G. lemaneiformis* was continuous, without introns. This is different from human dismutase where the SOD gene contains five exons and four introns [21]. The Mn-SOD of *Porphyra yezoensis* contained four exons and three introns [17]. This accounts for the fact that genomic DNA for the Mn-SOD gene of *G. lemaneiformis* was much smaller than that of *Porphyra yezoensis*.

### 2.3. Southern Blot

**Figure 4:**
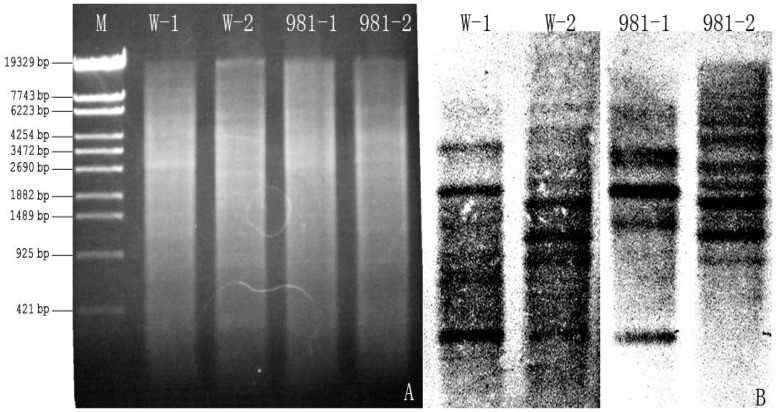
Genomic DNA digested with endonucleases and Southern blotting of Mn-SOD both in W and 981. W-1 and 981-1 were digested with SacI and KpnI, W-2 and 981-2 were digested with XbaI and NdeI.

Southern blot analysis was performed to characterize the genomic organization of Mn-SOD in *G. lemaneiformis*. The genomic DNA was completely digested with endonucleases for hybridization. W-1 and 981-1 were digested with SacI and KpnI, W-2 and 981-2 were digested with XbaI and NdeI. More than one band appeared on the membrane (Figure 4). The result showed that the Mn-SOD related sequences existed as multiple copies, possibly four, in *G. lemaneiformis*.

### 2.4. Quantitative Analysis of Mn-SOD mRNA Transcription and SOD Activity Assay

**Figure 5:**
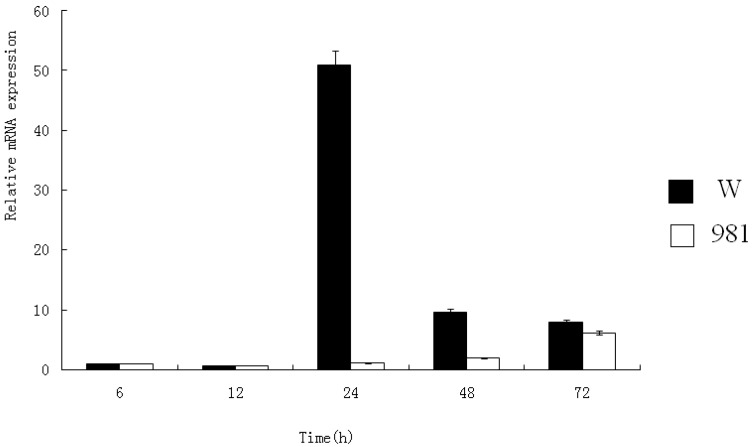
Quantitative analysis of Mn-SOD mRNA transcription in W and 981 at 32 °C. Each datum represents the mean of three determinations on the same sample with standard deviation.

Mn-SOD mRNA transcription in both the wild and cultivar 981 strains was significantly affected by high temperature; however, their patterns of change were quite different (Figure 5). W-SOD transcription increased sharply at 24 h, followed by a substantial decrease. 981-SOD transcription increased gradually from 24 h to 72 h, with no spike at 24 h. These results showed that Mn-SOD was induced under heat stress. In the wild type, the transcription of Mn-SOD began earlier and the levels were higher. With extended stress time, W-SOD transcription noticeably decreased while the 981-SOD transcription continued to increase. This suggests that in *G. lemaneiformis*, as elsewhere, Mn-SOD activity prevents or lessens the harm caused by heat stress, resulting in greater high temperature tolerance.

**Figure 6:**
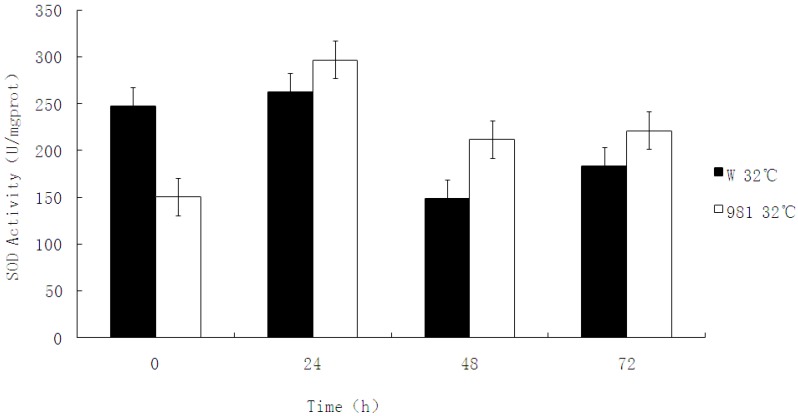
SOD activity assay of W and 981 at 32 °C. Each datum represents the mean of three determinations on the same sample with standard deviation.

SOD activity under high temperature stress was assayed. Figure 6 shows that without high temperature stress (0 h), the SOD activity of the wild strain was higher than that of cultivar 981. However, under temperature stress, the SOD activity of 981 increased over that of the wild strain at all time points. This result shows that cultivar 981 can actively respond to heat stress and suggests that it removes ROS effectively to help overcome the detrimental effects of high temperature.

A comparison was made of SOD activity and mRNA transcription of the wild strain and cultivar 981 under heat stress. Although the wild strain transcribed more mRNA than cultivar 981 at 24 h, SOD activity assays showed that the SOD activity of cultivar 981 was stronger than that of the wild isolate. This suggested that the wild type was more sensitive to heat stress than cultivar 981, and responded strongly at the mRNA expression level. However, gene expression is not only determined by the transcription level, but also the stability and translation level of mRNA [22]. In the wild plants, mRNA may not be as stable under heat stress as that of cultivar 981. Alternatively, cultivar 981 may translate mRNA more efficiently than the wild isolate at the higher temperature. The activity of Mn-SOD in cultivar 981 was higher than that of the wild isolate. With extended stress time, W-SOD transcription decreased apparently while the 981-SOD transcription increased gradually. Activity of 981-SOD was higher than the wild-SOD from 24 h to 72 h under heat stress. This suggests that Mn-SOD activity is directly related to its heat tolerance.

## 3. Experimental

### 3.1. Materials

Cultivar 981 of *G. lemaneiformis* was collected from a cultivation field at Nanao Island, Shantou, China. The wild isolate of *G. lemaneiformis *was obtained from Zhanshan Bay, Qingdao, China. The algae were thoroughly rinsed with sterilized seawater and cultured in sterilized natural seawater supplemented with 100 μM NaNO_3_ and 10 μM NaH_2_PO_4_•H_2_O (final concentration) at 20 ± 1 °C. Intensity of illumination was 50–60 μmol photon/m^−2^·s^−1^ with a light/dark period of 12:12 h.

### 3.2. RNA Extraction and cDNA Synthesis

Total RNA was extracted from 200 mg fresh *G. lemaneiformis* using RNAprep Pure Plant Kit (Tiangen, Beijing, China) according to the manufacturer’s protocol. The RNA was treated with DNaseI for 15 min in order to remove the remaining genomic DNA. The first strand of cDNA was synthesized with 2 µg of purified total RNA using the PrimerScript™ RT-PCR Kit (Takara, Dalian, China) according to the instruction manual.

### 3.3. Cloning of the Full-Length Mn-SOD cDNA

**Figure 7:**
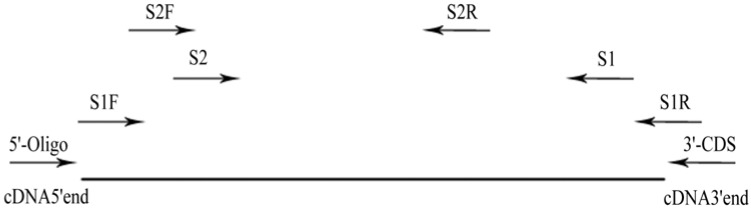
The position of primers used for PCR ampliﬁcation.

We initially obtained partial Mn-SOD cDNA sequences from the SSH libraries of cultivar 981 and the wild isolate under heat stress. The 5' end of Mn-SOD was amplified using the S1 primer and 5'-Oligo primer. The PCR program was 94 °C for 5 min followed by 35 cycles, at 94 °C for 30 s, at 55 °C for 30 s, at 72 °C for 1 min and a final extension step at 72 °C for 5 min. The 3' end of Mn-SOD was amplified using the S2 primer and 3' CDS primer. The PCR amplification was conducted as one cycle of 95 °C for 5 min followed by 35 cycles of 94 °C for 45 s, 55–65 °C for 30 s, 72 °C for 1 min and a final extension step at 72 °C for 5 min. The PCR products of both W and 981 were purified by 2% agarose gel, cloned into pMD18-T vectors, transformed into competent *E. coli *DH5α cells and then the positive recombinants (3 clones were selected for each sequence) were screened for sequencing. The primer sets utilized in this study are listed in Table 1 and the positions of primers are marked in Figure 7.

Full-length Mn-SOD cDNA sequences for wild plants and cultivar 981 were obtained by overlapping three cDNA fragments, the 5' end, 3' end and core region of the Mn-SOD cDNA. The ORF was analyzed and the corresponding amino acid sequences were deduced from the nucleotide sequences using DNAMAN software. Similarity analysis was performed with the BLAST program [19]. Alignment of multiple sequences was performed by Clustal X1.81.

**Table 1:** Nucleotide sequences of primer pairs used for PCR ampliﬁcation.

Primer Name	Sequences (5'→3')
S1	CCCAGACGTCCAAACCAAGGATCGGGG
S2 5'-Oligo 3'-CDS	GGACCCTCATGGCCAAGCCCGGATCCGC AAGCAGTGGTATCAACGCAGAGTACGCGGG AAGCAGTGGTATCAACGCAGAGTACTTTTTTTTTTTTTTTTVN
S1F	ATGAATTTCGCCCTCCCCGCTCTC
S1R	TTACGCGACAGGGGTGTCGAAGAC
S2F	CATCCAGGCAAACATCCC
S2R	GGCTTTGACGGAACCCAT
18sF	TGGTGGAGTGATCTGTCTGGTT
18sR	TTGGCCCGTTCAGTGTAGC

### 3.4. Cloning of the Mn-SOD DNA Sequence

Genomic DNA was extracted from cultivar 981 and wild plants using the Universal Genomic DNA Extraction Kit (Takara). Primers S1F (begin with start codon ATG) and S1R (begin with TTA which complements with stop codon TAA) were designed according to the cDNA sequences. DNA sequences were amplified by using genomic DNA as a template. The PCR program was 94 °C for 5 min followed by 35 cycles, at 94 °C for 30 s, at 54 °C for 30 s, at 72 °C for 1 min and a final extension step at 72 °C for 5 min. The PCR products were purified and sent to the Invitrogen Company (Shanghai, China) for sequencing.

### 3.5. Genomic Southern Blot

DNA from the wild and cultivar 981 strains (2.5 μg) was digested with two endonuclease mixtures (each 0.5 U): Sac I and Kpn I, Xba I and Nde I. The fragments were separated in a 0.8% agarose gel, and then transferred onto a nylon membrane. The SOD DNA fragments of the wild isolate and cultivar 981 were then labeled by DIG to be used as probes. The procedure followed for hybridization and exposure was according to the DIG High Prime DNA Labeling and Detection Starter Kit I (Roche, Mannheim, Germany) for Southern blot analysis.

### 3.6. Quantitative Analysis of Mn-SOD mRNA Transcription

Real-time PCR analysis of the Mn-SOD mRNA transcription at high temperature (32 °C) was performed using RealMaster Mix (SYBR Green) (Tiangen) and an ABI 7500 FAST real time PCR platform, at time points from 6 h to 72 h. Mn-SOD specific primers—S2F, S2R and primers 18sF, 18sR for the 18S rRNA gene (EU561239.1) were used for the real-time PCR. The 18S rRNA was used as an internal control. The cycle parameters consisted of one 2 min cycle at 95 °C and then 40 cycles of 15 s at 95 °C, 45 s at 60 °C followed by 30 s at 68 °C. Data were collected at the end of each extension step. The relative quantities of gene transcripts for the treatment groups were analyzed by the 2^−ΔΔCt^ method [23].

### 3.7. SOD Activity Assay

SOD activities were assayed when the *G. lemaneiformis* had been subjected to heat stress of 32 °C for 0 h, 24 h, 48 h and 72 h. The crude enzyme extracts were prepared using 0.5 g samples in 5 mL extraction buffer (0.1 mol L^−1^ phosphate buffer, pH 7.5). The homogenate was centrifuged at 1,000 g for 10 min at 4 °C and the supernatants were then used for the enzyme assays. SOD activity was estimated according to the instruction manual of the SOD Detection Kit (Nanjing Jiancheng Bioengineering Institute, Nanjing, China). This assay for SOD activity with xanthine-xanthine oxidase used as a superoxide anion radical generator involves inhibition of oxidation of hydroxylamine hydrochloride to nitrite, which is purple when chromogenic reagent is added. The SOD can catalyse the dismutation reaction of superoxide anion radical and reduced the superoxide anion radical, so the amount of nitrite will decrease and the purple color will be lighter. Measured absorption spectrophotometric values both of SOD sample tube (S) and the control tube (C) at 550 nm wavelength were used to calculate the activity of SOD according to the following formula:

SOD activity (*U/mgprot*) = (C–S)/C ÷ 50% × times for dilution of reaction system ÷ protein content in tissues (mgprot/mL)

One unit of SOD is defined as the amount of the enzyme in per mg tissue protein of 1 mL sample solution that inhibits the oxidation reaction of hydroxylamine hydrochloride to nitrite with superoxide anion by 50%.

### 3.8. Statistical Analysis

Data in Section 3.6 and Section 3.7 were analyzed using Student’s *t*-test for two-tailed arrays using the statistical programme in Microsoft Excel, Version 7.0.

## 4. Conclusions

Our analysis suggests a direct relationship between SOD activity and heat resistance in *G. lemaneiformis*. This research may provide insights for selecting new, more heat tolerant cultivars of *G. lemaneiformis*. 
